# 634. Head-to Head Comparison of the Performance of BIOFIRE^®^ SPOTFIRE^®^ Respiratory/Sore throat Panel, Cepheid Xpert^®^ Xpress SARS-CoV-2/Flu/RSV and Cobas^®^ SARS-CoV-2 & Influenza A/B Assay for Detection of Respiratory Viruses

**DOI:** 10.1093/ofid/ofad500.700

**Published:** 2023-11-27

**Authors:** Dithi Banerjee, Anjana Sasidharan, Stephanie Gummersheimer, Sydnie Petty, Amanda M Hayes, Minati Dhar, Rangaraj Selvarangan

**Affiliations:** Children's Mercy Hospital, Kansas City, Missouri; Childrens Mercy Hospital, Missouri, Kansas; Children's Mercy Hospital, Kansas City, Missouri; MCC-Longview, Raytown, Missouri; Children's Mercy Hospital, Kansas City, Missouri; Children's Mercy Hospital, Kansas City, Missouri; Children’s Mercy Kansas City, Kansas City, Missouri

## Abstract

**Background:**

Several multiplex assays have been developed for the detection of respiratory pathogens. We compared the performance of BIOFIRE**^®^** SPOTFIRE**^®^** Respiratory/Sore Throat (R/ST) Panel (BioMérieux) with Xpert® Xpress SARS-CoV-2/Flu/RSV assay (Cepheid) and Cobas® SARS-CoV-2 & Influenza A/B assay (Roche) for the detection of Flu A, Flu B, SARS CoV-2 and RSV from nasopharyngeal swabs (NPS).

**Methods:**

Total of 250 leftover NPS (50 each for Flu A, Flu B, RSV, SARS-CoV-2 and negatives) obtained from children as a part of routine diagnostic care and tested previously by standard of care assay were enrolled in the study and tested on all three respiratory panel multiplex platforms. Results for Flu A, Flu B and SARS CoV-2 were compared to a composite reference standard (consensus positive = positive result in ≥2 assays and consensus negative = negative result in ≥2 assays) to calculate positive percent agreement (PPA) and negative percent agreement (NPA). For RSV, PPA and NPA were calculated by comparing Xpert and SPOTFIRE assays only. Discrepant samples were tested by CDC single plex PCR for each of the viruses.

**Results:**

PPA and NPA for all targets is shown in Table 1. PPA for Flu A and Flu B were similar across all platforms and NPA >95% by all assays. PPA and NPA for RSV between Xpert and SPOTFIRE was 98% and 92% respectively. CDC PCR assays performed on samples with discrepant results confirmed 3/13 Flu A, 24/33 SARS-CoV-2 and 9/15 RSV detections with 50% samples being missed with CT < 35. SPOTFIRE can detect 15 respiratory pathogens (including viruses and bacteria) and was positive for a virus in 220/250 (88%) samples. Additional viral targets (excluding Flu A/B, SARS-CoV-2 and RSV) were detected either as single or co-detections (Figure 1). All samples with initial invalid results on Xpert (5, 2%), SPOTFIRE (3, 1.2%) and Cobas (2, 0.8%) were valid on re-testing. Total hands-on time (specimen processing and loading, retrieving results) ranged between 1.13 minutes to 2.34 minutes for all assays.

Table 1.

**Figure:**
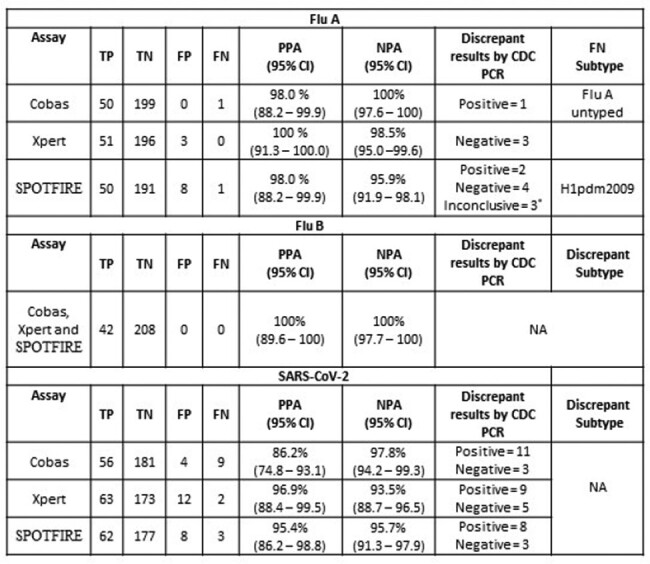

Performance characteristics of SPOTFIRE Respiratory/Sore Throat (R/ST) Panel, Xpert® Xpress SARS-CoV-2/Flu/RSV assay and Cobas® SARS-CoV-2 & Influenza A/B assay for detection of Flu A, Flu B and SARS-CoV-2 *Samples tested negative for universal Flu A PCRs but positive for Flu A typing PCRs with >37 CT. NA – Not Applicable, TP – True positive, TN – True Negative, FP – False Positive, FN - False Negative

Figure 1.

**Figure:**
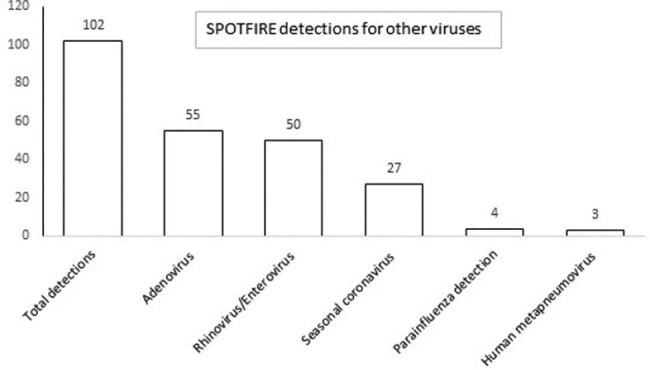

Detections of other respiratory viruses (excluding Flu, SARS-CoV-2 and RSV) on the SPOTFIRE platform

**Conclusion:**

All 3 assays showed comparable performance ( >95% agreement) for Flu A and B detection; performance for SARS-CoV-2 and RSV detection varied, even for samples with high viral load. SPOTFIRE provides the advantage of detecting many respiratory pathogens. Overall, the assays were easy to perform with minimum technical skill.

**Disclosures:**

**Rangaraj Selvarangan, BVSc, PhD, D(ABMM), FIDSA, FAAM**, Abbott: Honoraria|Altona Diagnostics: Grant/Research Support|Baebies Inc: Advisor/Consultant|BioMerieux: Advisor/Consultant|BioMerieux: Grant/Research Support|Bio-Rad: Grant/Research Support|Cepheid: Grant/Research Support|GSK: Advisor/Consultant|Hologic: Grant/Research Support|Lab Simply: Advisor/Consultant|Luminex: Grant/Research Support

